# ﻿Hybrid assembly of *Penicilliumrubens* genomes unveils high conservation of genome structural organisation and the presence of Numts in nuclear DNA

**DOI:** 10.3897/imafungus.16.145175

**Published:** 2025-05-23

**Authors:** Elena Requena, Javier Veloso, Eduardo A. Espeso, Inmaculada Larena

**Affiliations:** 1 Grupo Hongos Fitopatógenos, Departamento de Protección Vegetal, Instituto Nacional de Investigación y Tecnología Agraria y Alimentaria, Consejo Superior de Investigaciones Científicas (INIA-CSIC), Madrid, Spain Instituto Nacional de Investigación y Tecnología Agraria y Alimentaria, Consejo Superior de Investigaciones Científicas (INIA-CSIC) Madrid Spain; 2 Departamento de Biología Funcional, Escuela Politécnica Superior de Ingeniería, Universidad de Santiago de Compostela, Lugo, Spain Universidad de Santiago de Compostela Lugo Spain; 3 Laboratorio de Biología Celular de Aspergillus, Departamento de Biociencias Celulares y Moleculares, Centro de Investigaciones Biológicas Margarita Salas-CSIC, Madrid, Spain Centro de Investigaciones Biológicas Margarita Salas-CSIC Madrid Spain

**Keywords:** Biocontrol agent, Numts, *Penicilliumrubens*, PO212, S27, whole genome sequencing PacBio

## Abstract

The search for highly accurate chromosomal reference genomes has become a primary objective for the fungal research communities. Various genomic events, including insertions, deletions, inversions and movement of transposable elements, can modify the genomic architecture, resulting in chromosomal rearrangements. Long sequence reads enhance the accuracy and reliability of the assembly procedure, facilitating the study of these genomic characteristics. Here, we have utilised a combination of PacBio and Illumina sequencing technologies to generate hybrid assemblies of *Penicilliumrubens* strains 212 (PO212) and S27. These assemblies were then subjected to a comparative analysis in order to elucidate the chromosomal rearrangements that underpin the observed genomic differences, with a particular focus on their implications in the biocontrol phenotype against phytopathogenic fungi. This approach has enabled us to obtain the assembly of both PO212 and S27 genomes, with each organised into 13 scaffolds. The genomic organisation between these two isolates is highly conserved and the presence of transposable elements between the strains does not reveal major differences. Using the hybrid assemblies, we were able to detect, for the first time in the genus *Penicillium*, the presence of two nuclear mitochondrial DNA segments (Numts) in the genomes of the PO212 and S27 strains. The differences in biocontrol phenotype displayed by PO212 and S27 strains are independent of their genome organisation. These genomes provide new information for the existing database repositories.

## ﻿Introduction

The last few decades have seen a technological evolution in which new sequencing methods have been developed very rapidly, making the sequencing and subsequent assembly processes easier, faster and at lower costs, with a major impact on various fields of research (Satam et al. 2023). The development of these new methods has allowed the sequencing of isolates of interest, with the objective of identifying the genetic basis for interesting phenotypes. This has led to improvements in the identification process and subsequent taxonomic reclassification. An example of this is the case of *Penicilliumrubens* and *Penicilliumchrysogenum* strains. There is a controversy surrounding the differentiation of these two species of *Penicillium* (Houbraken et al. 2011) because of the complexity of making taxonomic classification, based on morphological descriptions (Visagie et al. 2014). The first strain of either species to be fully sequenced was *P.chrysogenum* Wisconsin 54-1255 (GCA_000226395.1) (van den Berg et al. 2008), which was reclassified as *P.rubens* years later (Houbraken et al. 2011). This reclassification also affected the strain P2niaD18 (Houbraken et al. 2011; Specht et al. 2014), a descendant from the semi-industrial penicillin producer strain *P.chrysogenum* NRLL1951 (Martín 2020). The strain 212 of *P.rubens* (PO212, ATCC201888), previously classified as *Penicilliumoxalicum*, was also reclassified. This strain has gained importance for its biocontrol activity (BA) against different pathogens in various horticultural crops (De Cal et al. 1995, 2008, 2009; Larena et al. 2003; Martínez-Beringola et al. 2013). As BA does not appear to be a prevalent characteristic of all *P.rubens* strains, PO212 was selected for further characterisation due to its ability to induce resistance in plants (De Cal et al. 1999). This phenotypic characteristic has prompted a comprehensive investigation of the genome of the PO212 strain, with a focus on comparative analysis with S27, a soil isolate of *P.rubens* lacking BA (Requena et al. 2023a).

In recent years, an increasing number of genomes have undergone large-scale analyses; as a result, the number of genomes from these species deposited at NCBI now exceeds 100, albeit with different levels of assembly quality. One of the best-assembled genomes is that of P2niaD18 (GCA_000710275.1), which is organised into five scaffolds. Consistent with the four chromosomes of *Penicilliumnotatum* and *P.chrysogenum* observed in the pulsed field electrophoresis (Fierro et al. 1993), the four largest scaffolds correspond to the nuclear DNA/chromosomes and the fifth corresponds to the mitochondrial genome (Specht et al. 2014). As more strains of this clade have been subjected to whole genome sequencing, genomic organisation studies have been carried out in comparison to the P2niaD18 or Wisconsin 54-1255 genome assemblies (Wang et al. 2014; Pathak et al. 2020). In a previous study, the genomes of strains PO212 and S27 of *P.rubens* were sequenced and compared (Requena et al. 2023a). This study revealed a high conservation of sequence between the two strains; however, the assemblies obtained did not allow the study of structural reorganisations and repeated elements that their genomes might have undergone.

The genomic organisation of microorganisms is subject to continuous changes, as is the integration of both exogenous and endogenous DNA. The process by which exogenous DNA is integrated into the nucleus is known as horizontal gene transfer (HGT). The phenomenon of HGT has been observed from bacteria to fungi (Gutiérrez et al. 1999; van den Berg et al. 2008) and between fungi (Slot and Rokas 2011). In fungi, another source of DNA for integration may be the formation of heterokaryons, whose vegetative incompatibility may lead to degradation of the resident or invading nuclei and its nuclear DNA (Marek et al. 2003). Fragments of this partially degraded genomic DNA may integrate into the remaining nucleus and constitute a source of genetic variation. The ability of these organisms to integrate DNA into their genomes has been widely exploited by researchers to integrate exogenous DNA for genetic studies in many fungal species, such as the model fungus *Aspergillusnidulans* (Tilburn et al. 1983) and more recently in *P.rubens* (Requena et al. 2023b).

The origin of new DNA fragments inserted into the nuclear genome is not always extracellular. Two other cases that add variability to genome organisation are the movement of Transposable Elements (TEs) in the genome and the insertion of endogenous DNA from organelles, such as mitochondrial DNA. The latter category is known as Numts (nuclear mitochondrial DNA segments). López et al. (1994) coined this term. Van den Boogaart et al. (1982) described the presence of the same gene in the nuclear genome of *Neurosporacrassa* and in the mitochondrial genome of *Saccharomycescerevisiae*. Numts have been identified in several species, including fungi, plants, mammals and insects (López et al. 1994; Noutsos et al. 2005; Pamilo et al. 2007; Sacerdot et al. 2008; Trian and DeWoody 2008; Li et al. 2021). Hazkani-Covo et al. (2010) presented a study on the presence of Numts in 85 fully-sequenced eukaryotic genomes and concluded that Numts are common in all groups studied. Amongst fungi, the highest Numt content was found in *Phaeosphaerianodorum* SN15 with 77.142 Kb (Hazkani-Covo et al. 2010). However, to our knowledge, the presence of Numts in the genus *Penicillium* has not been described. The most accepted hypothesis for the generation of Numts is via the degradation of abnormal mitochondria during mitophagy (Campbell and Thorsness 1998). It has been suggested that double-strand breaks (DSBs) by the non-homologous end joining (NHEJ) machinery are required for mitochondrial DNA entry and integration into the nucleus (Blanchard and Schmidt 1996). In contrast, transposable elements are a well-known source of variability in the structure of a genome. Eukaryotic genomes have a high content of repetitive sequences as TEs that need to be identified. Large rather than short genomic sequences facilitate the identification of TEs. Transposable elements cause small deletions or insertions and even induce translocations (Bourque et al. 2018). TEs are a source of mutations, as they are associated with genomic rearrangements and the insertion and deletion of TEs is imprecise (Bourque et al. 2018). This may alter the expression of surrounding genes, amongst other effects.

In conjunction with the analysis of structural genomic rearrangements in a genome, it is interesting to examine genes encoding members of large and well-characterised protein families, such as the Carbohydrate-Active enZymes (CAZymes) (Barrett et al. 2020). In addition to genome variability studies, this group of enzymes reflects the amount and diversity of carbohydrates and glycoconjugates found in nature (Cantarel et al. 2009). Furthermore, with the expansion of genomic data, it is anticipated that the number of CAZymes will continue to rise (Garron and Henrissat 2019), a development that will be of great benefit to the completion of genomic variability studies using this database, as well as the advancement of knowledge of the microorganism´s capacity to produce specific enzymes.

In order to increase the knowledge of structural genomic rearrangements in PO212 compared to S27, we performed PacBio and Illumina sequencing and subsequent hybrid assemblies of the genomes of both strains of *P.rubens*. These new data provide the basis for comparing structural variations in PO212. Using different strategies, we underscored the genetic similarity in these assemblies, by analysing a large and conserved family of proteins and the presence and number of transposable elements. Notably, we identified two mitochondrial DNA insertions in the nuclear genome in this fungal species.

## ﻿Materials and methods

### ﻿Strains and growth conditions

The *P.rubens* strains, including PO212 and S27, isolated from diverse agricultural soils and plant samples in Spain, are listed in Table 1 (Villarino et al. 2016; Espeso et al. 2019; Requena et al. 2023a). Conidia from these strains were long-term stored in 20% glycerol at -20°C. In order to obtain the inoculum, *Penicillium* strains were grown on potato dextrose agar (PDA, Difco) and incubated at 25°C for a period of five days. For short-term storage, strains were maintained at 4°C on PDA.

**Table 1.** List of *Penicilliumrubens* strains used in this study.

Strain	Origin	Host	BA ^†^	Reference
**PO212**	Spain	Soil	+	De Cal et al. (1995)
**S27**	Spain (Ávila)	Soil	-	Villarino et al. (2016)
**S17**	Spain (Segovia)	Soil	-	Villarino et al. (2016)
**S71**	Spain (Segovia)	Soil	-	Villarino et al. (2016)
**S73**	Spain (Segovia)	Soil	+	Villarino et al. (2016)
**CH2**	Spain (Madrid)	Leaf of a perennial plant	- ^‡^	Requena et al. (2023a)
**CH5**	Spain (Madrid)	Shoot of a perennial plant in a field of peach trees	- ^‡^	Requena et al. (2023a)
**CH6**	Spain (Madrid)	Deep soil sample in the field of peach trees	+ ^‡^	Requena et al. (2023a)
**CH8**	Spain (Madrid)	Shoot of a perennial plant in a pine forest	+ ^‡^	Espeso et al. (2019)
**CH16**	Spain (Lérida)	Outbreak of pruning shoot	+ ^‡^	Requena et al. (2023a)

^†^BA: Biocontrol Activity. ^‡^ Unpublished results, general screening of BA of strains from INIA-CSIC collection. The symbol + indicates BA. The symbol – indicates no BA.

### ﻿DNA extraction and sequencing

For total DNA extraction of PO212 and S27 strains, cultures were grown in liquid minimal medium (MM; Espeso et al. (2019)) supplemented with D-glucose 1% (w/v) and 5 mM ammonium tartrate as carbon and nitrogen sources, respectively. The cultures were then incubated at 25°C for 2 days. Mycelium was harvested by filtration through Miracloth (Calbiochem, Merck-Millipore, Darmstadt, Germany). Samples were lyophilised for at least 6 hours and pulverised using ceramic beads in a FastPrep-24 homogeniser (MP Biomedicals™) with a single 20-seconds pulse at a power setting of four. Genomic DNA extraction procedure followed the protocol described by Requena et al. (2023a).

Macrogen (Korea) performed PacBio and Illumina sequencing. Integrity of genomic DNA was analysed and DNA libraries were constructed on SMRT (single molecule real time sequencing) Library (20 kb). Sequencing procedure was carried out on SMRT Cell Run in Sequel I. For error correction, library construction was performed with Nextera DNA XT and sequencing in an Illumina NovaSeq6000 platform using 150 base pairs (bp) paired-end sequencing reads. The DNA sequencing data analysed in this study were deposited at the National Center for Biotechnology Information (NCBI) under BioProject PRJNA887566 Assembly and Scaffolding.

### ﻿Assembly and scaffolding

Reads obtained from sequencing were subjected to quality analysis before proceeding with the assemblies. The assemblies of PO212 and S27 genomes were conducted using Flye (v.2.4.2) software, employing the default parameters (minimum overlap auto (3k–10k), with a 20% error, Haplotype mode activated, one polishing by default) (Kolmogorov et al. 2019). After initial assembly, the Illumina reads were mapped using Pilon (v.1.21) (Bruce et al. 2014). Pilon was run three times using paired-end reads with the following parameters; --fix bases, baps, local --nostrays --mindepth 0.01. To evaluate structural changes, dot-plot analyses were performed using CLC Genomics Workbench 22.0.2 (QIAGEN Bioinformatics) with the “Whole Genome Alignment” plugin installed. The parameters used were 100 as minimum initial seed length and mismatches in seeds allowed. Integrative Genome Viewer (IGV version 2.9.4; Robinson et al. (2011)) and Apollo (Dunn et al. 2019) tools were utilised for visualising genomic data.

After initial assemblies were obtained and errors corrected using short reads from Illumina sequencing, a manual curation was performed to reduce the number of scaffolds while improving the assemblies using two different strategies. The first strategy consisted of the comparison of sequences of the 5’ and 3’ ends of scaffolds that were predicted to overlap, based on the information provided by the dot-plot. As an example, scaffolds PO212_1 (previously reverse complemented) and PO212_11 were joined following this strategy. The second strategy involved the search for overlapping regions between the sequences of the 5’ and 3’ ends of all scaffolds, despite the absence of evidence in the dot-plot. For instance, following this approach, we assembled scaffold PO212_3 with scaffold PO212_17. To be considered for merging, two scaffolds had to overlap at least 1,500 bp. Upon identifying overlaps, the fasta sequence files were manually reconstructed and specific oligonucleotides were designed to flank these reassembled regions.

In order to evaluate the quality of these manually merged regions, raw reads were mapped against each genome and the final assembled version of scaffolds was verified experimentally using PCR techniques. The oligonucleotides utilised in these amplifications are enumerated in Suppl. material 4. PCR was performed using repliQa HiFi ToughMix® (QIAGEN Beverly, Inc.) Quantabio with the following PCR conditions: 30 cycles of denaturation at 98°C/10 s and annealing at 68°C/1 s (for fragments ≤ 1 kb), 68°C/ 5 s/kb (for fragment between 1 ~ 10 kb) or 68°C/ 10 s/kb (for fragments ≥ 10 kb).

A Telomere Identification toolKit (tidk) was used to identify telomeric repeats (Brown et al. 2025) in the final assemblies. The *P.rubens* telomeric repeat was obtained from TeloBase. The module “tidk search” was used to search the genome using the known telomeric repeat, TTAGGGC, obtaining the counts of occurrence in windows across the genome. The window size to calculate telomeric repeat counts was the default of 10,000 bp.

In order to obtain the assembled mitochondrial genome for the PO212 and S27 strains, Illumina raw reads were mapped to the 27 kb P2niaD18 mitochondrial genome (GCA_000710275.1) (Specht et al. 2014). Samtools (Li et al. 2009) was the employed to extract the PO212 and S27 raw reads that had been mapped to the P2niaD18 mitochondrial genome. The A5-miseq pipeline (Coil et al. 2015) was subsequently used to assemble the reads into mitochondrial scaffold.

### ﻿Synteny and chromosome ordering

A comparison of PO212 and S27 assemblies for determining sequence similarity was conducted using CLC Genomics Workbench 22.0.2. Furthermore, sequence homology was determined using BLAST with a cut-off E value of 1E-10. The use of homologous loci facilitated the mapping of the relative positions and directions of scaffolds along the PO212 and S27 scaffolds. Sequences that exhibited at least 30 consecutive homologous loci were considered homologous regions. The coordinates of these homologous regions were then merged and flattened using a custom Python script and visualised using Circos v. 0.69 (Krzywinski et al. 2009). This script was also applied to assess the level of organisation of the PO212 genome with the P2niaD18 (GCA_000710275.1) (Specht et al. 2014) and Wisconsin 54-1255 (GCA_000226395.1) (van den Berg et al. 2008) reference genomes.

### ﻿Prediction of repetitive sequences

RepeatMasker version 4.0.7 (Smit et al. 2015), included in the REPET package (Jamilloux et al. 2017), was utilised to identify all repetitive elements by employing the fungal-specific repetitive sequence database from RepBase volume 29, issue 3 (girinst.org). Lower-scoring sequences (score < 400) were removed. Sequences overlapping at the same loci as other higher-scoring sequences were considered redundant and removed. Each repetitive element locus was grouped by class and counted for each of the two fungal strains.

### ﻿Gene predictions and functional annotation

To predict the genes contained within genome assemblies, the homology-based gene prediction programme, Gene Model Mapper (GeMoMa) (Keilwagen et al. 2016, 2018), was used. These predictions were trained using Augustus’ annotation files of the primary versions of the PO212 and S27 genomes (Requena et al. 2023a). RNA-Seq raw reads were added to GeMoMa to improve predictions of splicing sites in gene models. Nucleotide sequence conservation between the S27 and PO212 genes was assessed using BLASTn (Altschul et al. 1990); a threshold value below 1E-10 was used to identify homologous genes. CLC Genomics Workbench 22.0.2 was employed to examine single nucleotide variations (SNVs) between the genomes. Specifically, the Map Reads to Reference tool was utilised to map the reads, followed by the application of Local Realignment and Basic Variant Detection analyses. To detect changes in the coding region, the Filter Based on Overlap tool was applied and, finally, the Amino Acid Changes tool was applied to identify nucleotide changes leading to an amino acid change. The default parameters were used. After manual correction of the predicted genes, based on homology data with other strains, functional annotation of proteins from each genome was conducted using OmicsBox (formerly Blast2GO) (Götz et al. 2008). Functional annotation of proteins from the P2niaD18 genome was also performed to facilitate comparison with those from PO212 and S27. The proteins detected in the three genomes were classified into 25 functional categories using the COG (Clusters of Orthologous Group) classification within four groups: Metabolism, Cellular Processes and Signalling, Information Storage and Processing and Poorly Characterised. The identification of Carbohydrate-Active enZymes (CAZymes) was undertaken using the dbCAN 3 web server (Zheng et al. 2023), with the dbCAN CAZyme domain HMM database (E-value < 1e-15, coverage > 0.35) and the CAZy database (E-value < 1e-10^2^) serving as the reference databases. CAZymes identified by both databases were selected for further analysis.

### ﻿Phylogenetic analysis by Maximum Likelihood method

A phylogenetic tree was generated using the Maximum Likelihood method and the Tamura-Nei model with 1,000 replicates (Tamura and Nei 1993) by using MEGA11 software (Tamura et al. 2021). The percentage of trees in which the associated taxa clustered together is displayed next to the branches. The initial tree(s) for the heuristic search were automatically obtained by applying Neighbour-Joining and BioNJ algorithms to a matrix of pairwise distances estimated using the Tamura-Nei model and then selecting the topology with the highest log-likelihood value. The tree is shown to scale, with branch lengths measured as the number of substitutions per site. This analysis included 11-nucleotide sequences. Six of these corresponded to the alpha-(1,3)-glucanase (mutanase) encoding genes from PO212 (PO212g058370, PO212g085410, PO212g101870, PO212g096930, PO212g013760 and PO212g033560). Four corresponded to the alpha-(1,3)-glucanase encoding genes from P2niaD18 (KZN91491, KZN92410, KZN92305 and KZN94461). Furthermore, the sequence of a glycosyltransferase (PO212g014370) from PO212 was utilised as the root. The codon positions encompassed included the 1st+2nd+3rd+non-coding positions. The final dataset comprised 4060 positions.

### ﻿Abbreviations

**AA** Auxiliary activity

**BA** Biocontrol activity

**CAZymes** Carbohydrate-active enzymes

**COG** Clusters of orthologous group

**DSBs** Double-strand breaks

**ITS** Internal transcribed spacer

**MM** Minimal medium

**NHEJ** Non-homologous end joining

**ORF** Open reading frame

**PDA** Potato dextrose agar

**SNV** Single nucleotide variation

**TEs** Transposable elements

## ﻿Results

### ﻿Near chromosome-scale assembly of the PO212 and S27 genomes

**Figure 1.**
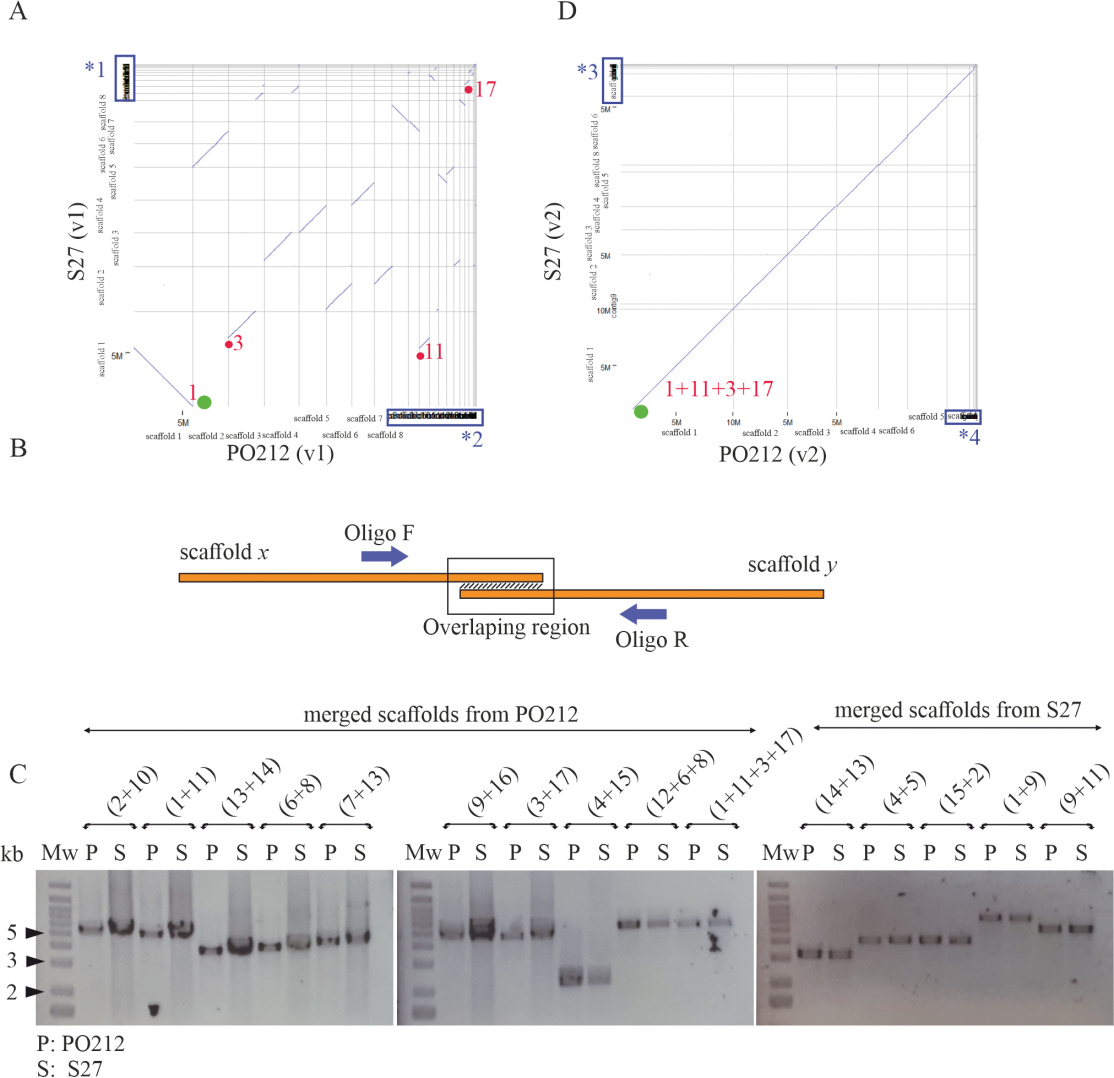
Manual curation of the PO212 and S27 assemblies: **A** whole-genome dot-plot between *P.rubens* strains 212 (PO212) and S27 using version 1 of the assemblies before manual curation. The PO212 assembly (*x*-axis) was utilised as the reference. Green circles mark the start of scaffold 1. Red circles indicate the start of the scaffolds designed for merging. The blue boxes on the *x*- and *y*-axis correspond to the following: *1: S27 scaffolds ordered from 9 to 20 and *2: PO212 scaffolds ordered from 9 to 23; **B** scheme of the overlapping region between the end of one scaffold and the start of another, together with the position of the designed oligonucleotides; **C** PCR product to verify the continuity of the regions in the PO212 and S27 strains. P indicates PO212 genomic DNA and S indicates S27 genomic DNA. The order from left to right is PO212 (2+10 = 5.5 kb, 1+11 = 5.3 kb, 13+14 = 4 kb, 6+8 = 4.3 kb, 7+13 = 4.9 kb, 9+16 = 5 kb, 3+17 = 4.9 kb, 4+15 = 2.3 kb, 12+6+8 = 5.6 kb and 1+11+3+17 = 5.5 kb). S27 (14+13 = 3.2 kb, 4+5 = 4.2 kb, 15+2 = 4.2 kb, 1+9 = 6.7 kb and 9+11 = 5.3 kb). Mw: Molecular weight marker. The black arrows indicate the band sizes of 5, 3 and 2 kb from top to bottom; **D** whole-genome dot-plot between strains PO212 and S27 using version 2 of assemblies after manual curation. The PO212 assembly (*x*-axis) was utilised as a reference. The green circle indicates the start of scaffold 1 in both genomes. The blue boxes on the *x*- and *y*-axis correspond to the following: *3: S27 scaffolds 7, 10, 11, 12 and 13 and *4: PO212 scaffolds 7, 8, 9, 10, 11, 12 and 13.

PacBio and Illumina technologies were utilised to sequence the genomes of *P.rubens* strains PO212 and S27, facilitating genomic analysis given the interest in the BA of PO212. The result of the automatic assembly process yielded 23 and 20 scaffolds, respectively (Fig. 1A). A subsequent manual assembly was then performed in order to reduce the number of scaffolds using the strategies mentioned in the Materials and Methods section. As an illustration of the manual reassembly process, we assembled PO212_1 (previously reverse complemented) and PO212_11 following the first strategy resulting in an overlap of 4,636 bp (Fig. 1B, Table 2). Following the second strategy, we assembled scaffold PO212_3 with scaffold PO212_17, yielding an overlap of 4,009 bp (Table 2). The new scaffolds formed by the joining of the 1+11 (PO212_1 and PO212_11) (first strategy) and 3+17 (PO212_3 and PO212_17) (second strategy), were then joined between the 3’ end of 1+11 and the 5’ end of 3+17, again following the first strategy. The first strategy involved the merging of the following scaffolds in the PO212 assembly: 1+11, 2+10, 4+15, 6+8, 7+13, 9+16, 13+14, 12+6+8 and 1+11+3+17. In the S27 assembly, we merged scaffolds 4+5 and 9+11. The second strategy involved the joining of scaffolds 3+17 in the PO212 assembly and 1+9, 15+2 and 14+13 in the S27 assembly. The experimental PCR approach permitted the validation of the genomic continuity of the scaffolds, which were linked by one of the two strategies (Fig. 1C). A dot-plot was generated, which revealed no structural rearrangements (Fig. 1D). This manual curation enabled the reduction of the number of scaffolds from both genomes to 13, the nomenclature, size and number of ORFs of which are described in Table 2. This represents a reduction in the number of scaffolds of approximately 43.5% and 35% for PO212 and S27, respectively.

**Table 2.** Data from hybrid assemblies followed by manual curation. Number of scaffolds, length and ORFs in the PO212 and S27 genomes are shown.

PO212					S27				
Manually merged scaffold ^†^	Overlapping regions (kb)^‡^	Final scaffold number	Size (bp)	N# predicted ORFs	Manually merged scaffold	Overlapping regions (kb)^‡^	Final scaffold number	Size (bp)	N# predicted ORFs
1+11+3+17	4.6/2.5/4.0	1	9,478,687	3,244	1+9+11	3.1/3.4	1	9,488,521	3,252
12+6+8	4.8/3.5	2	4,548,765	1,537	3 ^†^	-	2	4,123,581	1,391
2+10	4.7	3	4,107,425	1,403	4+5	1.8	3	2,083,268	717
15+4	1.6	4	3,513,604	1,211	6 ^†^	-	4	1,895,371	642
7+13+14	4.5/3.2	5	3,324,254	1,151	7 ^†^	-	5	2,933,455	1,019
5 **^†^**	-	6	2,396,790	822	8 ^†^	-	6	5,740,421	1,974
9+16	4.0	7	1,873,659	666	10 ^†^	-	7	1,884,646	671
18 **^†^**	-	8	355,628	133	12 ^†^	-	8	592,447	199
19 **^†^**	-	9	110,255	34	14+13	2.1	9	445,038	149
20 **^†^**	-	10	74,744	20	15+2	2.2	10	471,198	171
21 **^†^**	-	11	56,873	17	16 ^†^	-	11	128,065	44
22 **^†^**	-	12	47,614	18	17 ^†^	-	12	81,615	30
23 **^†^**	-	13	6,144	1	18 ^†^	-	13	74,708	20

^†^ Primary scaffolds not merged with other scaffolds. **^‡^** Overlapping regions between each pair of scaffolds.

### ﻿Genomic variations in PO212 and S27 assemblies

**Figure 2.**
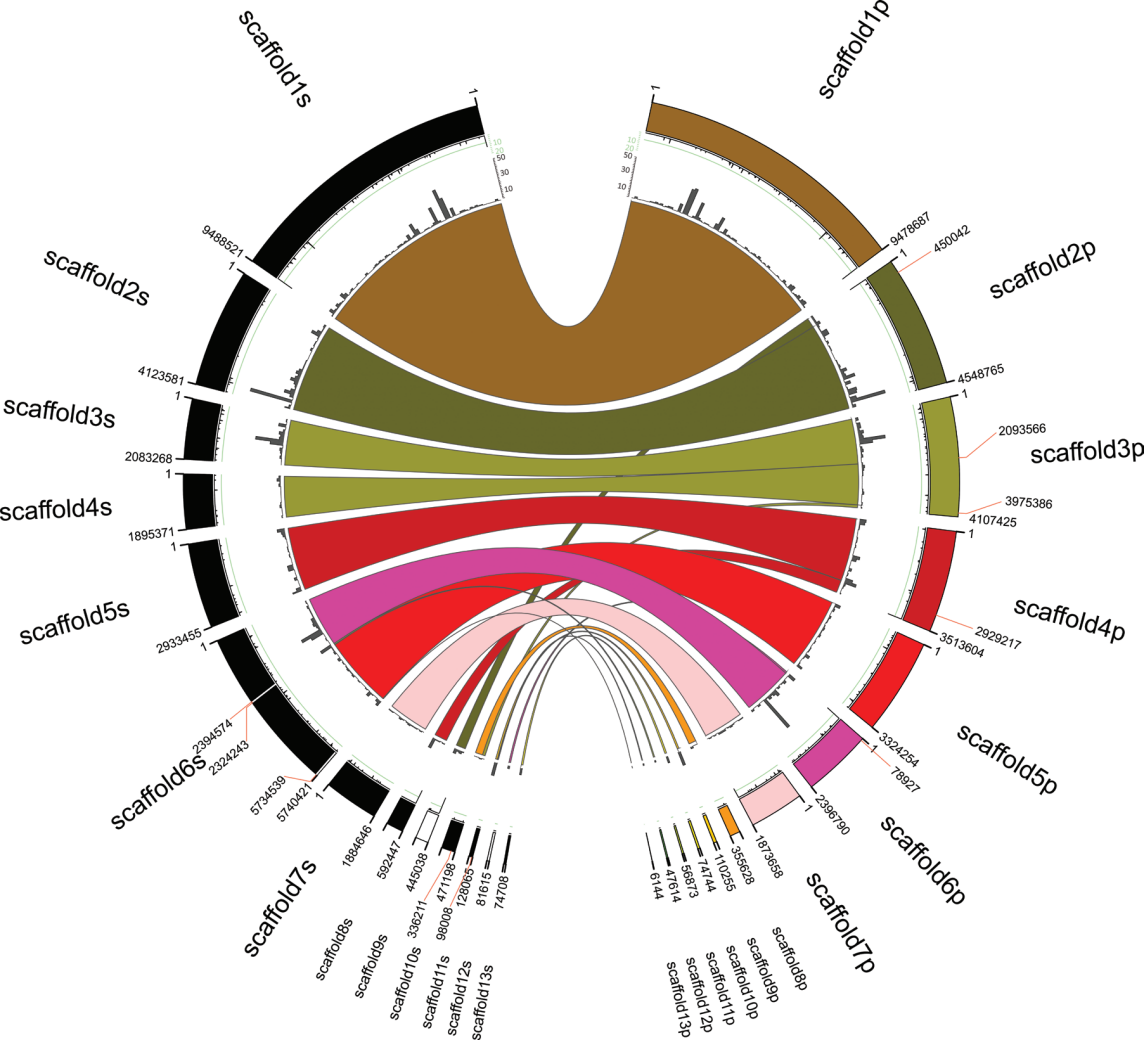
Synteny amongst the S27 genome (left) and the PO212 genome (right). The scaffolds corresponding to the PO212 strain have been delineated in 13 different colours on the right and are labelled as in the genome assembly. The external track, situated in close proximity to the scaffolds, displays the quantity of telomeric repeats TTAGGGC. The scale utilised for quantifying the number of repeats ranges from 0 to 20, with the green-coloured line denoting seven repeats in each telomeric repeat search window/range. The grey bars represent a track indicating the number of repetitive elements in 120 kb ranges. On the left, the S27 scaffolds are linked to the PO212 scaffolds by lines representing regions of high sequence similarity. High sequence similarity regions have at least 30 consecutive genes homologous to PO212. In black are labelled those S27 scaffolds whose nt sequences are in the same direction as the aligned PO212 sequence, while those in white are in the inverted direction as the aligned PO212 sequence. Numerical labels indicate the nucleotide of the break junction between rearranged regions.

Utilising the hybrid assemblies, we analysed the presence of differences in structural genomic organisation between both strains. Features, sizes and other data from the newly-assembled genomes and those of strains P2niaD18 and Wisconsin 54-1255 are shown in Table 3. There is a highly conserved genomic structure between the PO212 and S27 assemblies as shown by the synteny analysis (Fig. 2), with no translocations or inversions detected. The discontinuous regions or breakpoints observed correspond to regions with low sequence coverage or repetitions, which hinder the ability of the assembler to predict the genome more accurately. Moreover, it was observed that telomeric repeats are present in one of the terminal ends of scaffold 1, scaffold 2, scaffold 4, scaffold 6 and scaffold 7 for PO212. These sequences were detected at both terminal ends of scaffold 1 of S27, as well as at one of the terminal ends of scaffolds 8 and 9 of this strain assembly (Fig. 2).

**Table 3.** Genomic characteristics of the newly-sequenced strains PO212 and S27 together with P2niaD18 and Wisconsin 54-1255.

Assemblies	PO212	S27	P2niaD18	Wisconsin 54-1255
**GenBank code**	(GCA_027256995.2)	(GCA_027257005.2)	(GCA_000710275.1)	(GCA_000226395.1)
**Size (Mb)**	29.89	29.94	32.52	32.22
# **Scaffolds**	13	13	5	49
**Largest scaffold (Mb)**	9.47	9.48	13.59	6.38
**N50**	4.1	5.74	10.45	3.9
**L50**	3	2	2	4
**GC content** %	49,02	49.02	48.95	48.96
# **Gene**	10,257	10,279	11,839	12,943

### ﻿Comparative organisation of *P.rubens* genomes

**Figure 3.**
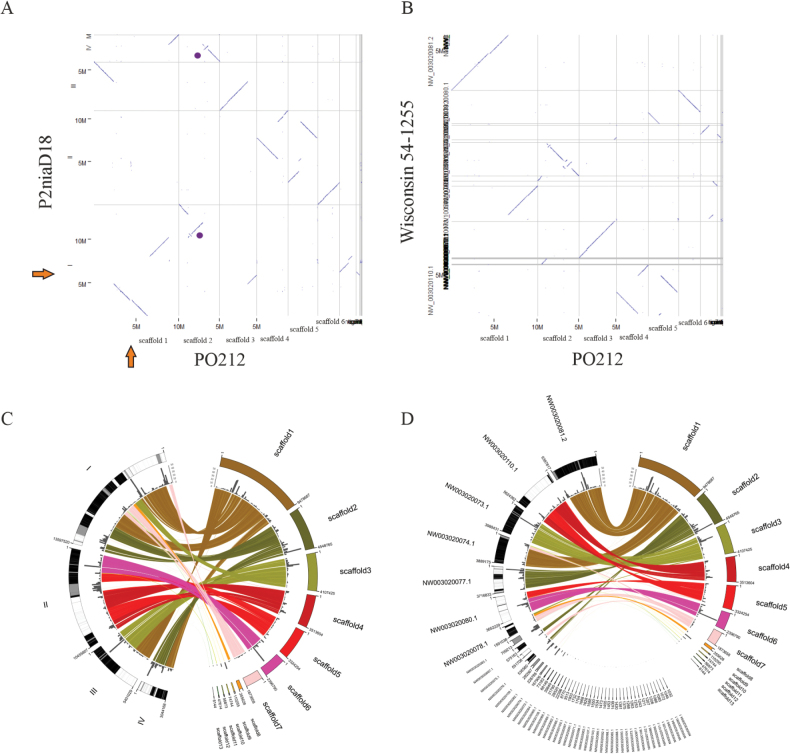
Synteny between the P2niaD18 genome (left) and the PO212 genome (right) (**A, C**) and between the Wisconsin 54-1255 genome (left) and the PO212 genome (right) (**B, D**): **A** whole-genome dot-plot between *P.rubens* strain 212 (PO212) and P2niaD18 genome (GCA_000710275.1). The PO212 assembly (*x*-axis) was utilised as a reference; **B** whole-genome dot-plot between *P.rubens* strain 212 (PO212) and Wisconsin 54-1255 genome (GCA_000226395.1). The PO212 assembly (*x*-axis) was utilised as a reference; **C** scaffold ordering of the P2niaD18 genome (left) to the scaffolds of the PO212 genome (right); **D** scaffold ordering of the Wisconsin 54-1255 genome (left) to the scaffolds of the PO212 genome (right). The PO212 scaffolds are illustrated on the right in 13 different colours, with the labelling corresponding to that employed in the genome assembly. The grey bars correspond to a track showing the number of repetitive elements in 120 kb ranges. On the left, the scaffolds of P2niaD18 (**C**) and Wisconsin 54-1255 (**D**) are linked to the PO212 scaffolds by lines representing regions of high sequence similarity. Numerical labels indicate the nucleotide of the break junction between rearranged regions.

In contrast to the conserved genomic structure between PO212 and S27, dot-plots of PO212-P2niaD18 and PO212-Wisconsin 54-1255 assemblies, showed notable differences at the level of organisation between them, indicating the presence of genomic rearrangements between these assemblies (Fig. 3). For example, segments of chromosome I of P2niaD18 are mainly distributed along scaffolds 1, 2, 3, 7 and 8 of PO212. Segments of scaffold 1 of PO212 were found in chromosomes I, III and IV of P2niaD18 (Fig. 3A, C). When analysing the comparison between PO212 and Wisconsin 54-1255 assemblies (Fig. 3B, D), we observed the occurrence of a general reorganisation of these genomes, with distinctive features, such as most of scaffold 2 of PO212 was found in scaffold NW003020077.1 in contrast to a major reorganisation in P2niaD18, where these sequences were distributed between chromosomes I and IV.

### ﻿Comparative analysis of gene predictions and protein annotation

**Figure 4.**
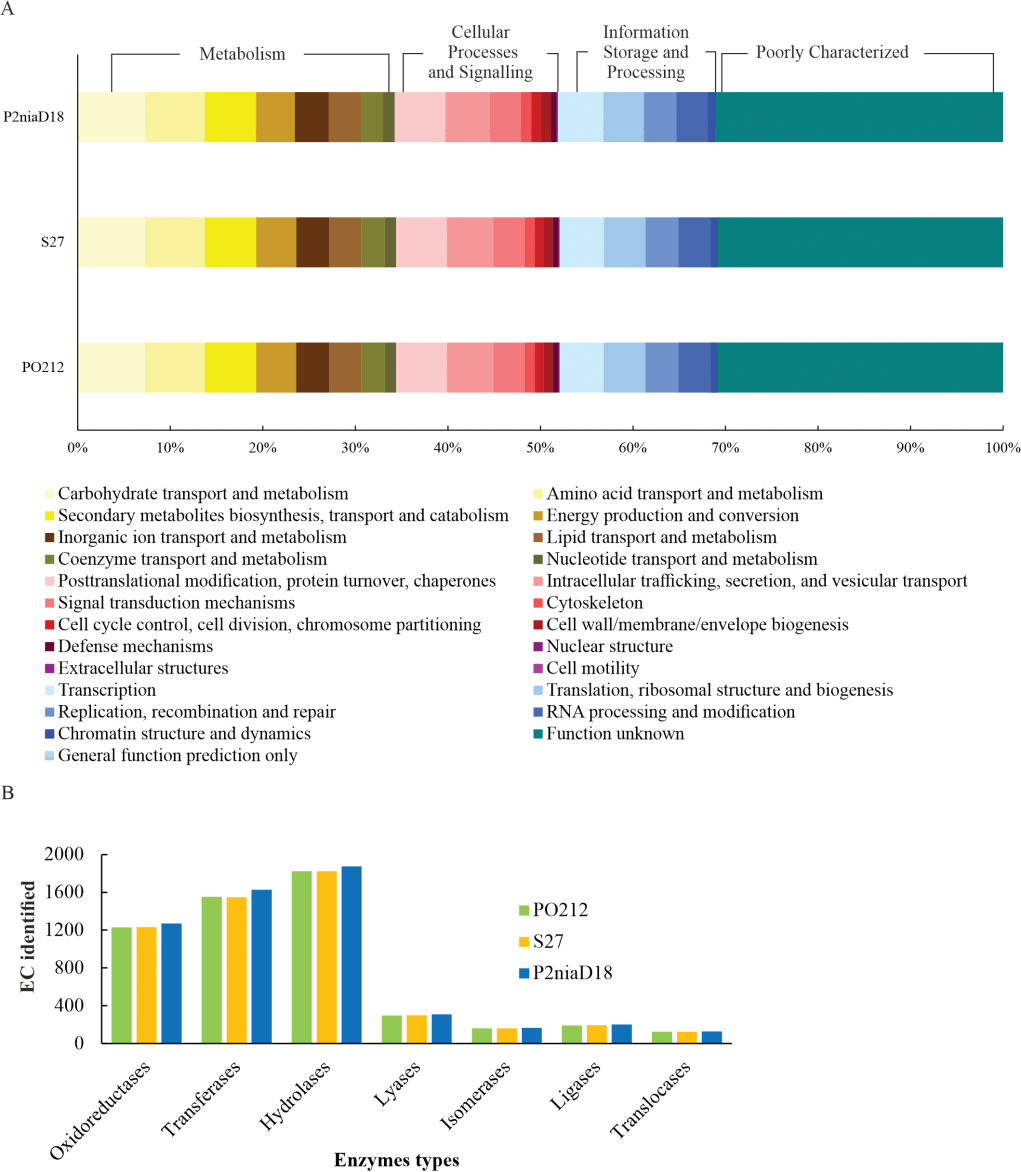
A comparative analysis of the identified proteins in the genomes of three *P.rubens* strains, PO212, S27 and P2niaD18, was conducted: **A** category abundance of proteins grouped into 25 functional categories using COG classification in PO212, S27 and P2niaD18; **B** enzyme distribution of identified proteins, based on EC class in the annotated genomes of the *P.rubens* strains PO212, S27 and P2niaD18.

In order to identify any discrepancies between the PO212 and the S27 assemblies, a search for such discrepancies was conducted using gene prediction and functional annotation of proteins through the utilisation of the OmicsBox platform. The predicted proteomes were organised into 25 clusters, which were subsequently classified into four main groups according to the distribution of the Clusters of Orthologous Group (COG) categories: Metabolism, Cellular Processes and Signalling, Information Storage and Processing and Poorly Characterised (Fig. 4A). The most represented protein groups in PO212 and S27 were Metabolism and Poorly Characterised (~ 66%). With regard to the three strains PO212, S27 and P2niaD18, the most represented groups in terms of enzyme type distribution were hydrolases, transferases and oxidoreductases (Fig. 4B), but no differences were found between PO212 and S27.

### ﻿Significant conservation in CAZymes arsenal amongst strains

The search for CAZymes was performed using the automated CAZymes annotation web server dbCAN 3 (Zheng et al. 2023). The results obtained from the PO212 and S27 databases demonstrated a high degree of similarity, with 407 and 406 members of this family identified in PO212 and S27 databases, respectively (Suppl. material 5). However, three discrepancies were identified (Suppl. material 6), which may be attributable to suboptimal predictions. The first difference corresponds to the final number of predicted CAZymes. This can be attributed to the absence of a distinctive domain in the glycosyl hydrolase (GH) protein encoded by S27g102640, as predicted by the dbCAN CAZyme domain HMM database. S27g102640 exhibits an identical nucleotide sequence to that of PO212g102060, which, in contrast to S27g102640, was predicted to be GH by both databases. The second discrepancy corresponds to the predictions of the dbCAN CAZyme domain HMM database, whose data differ between the gene models PO212g053760 and S27g052360. The presence of a highly repetitive nucleotide sequence may be the origin of the assembly variation between these two models. The final discrepancy observed between PO212 and S27 CAZymes predictions pertains to the signal peptide motif between PO212g088560 and S27g073030. The absence of such a prediction for S27g073030 was noted; however, genomic sequence analysis confirmed the presence of identical sequence for both loci, thereby providing evidence of a failure in motif prediction.

**Figure 5.**
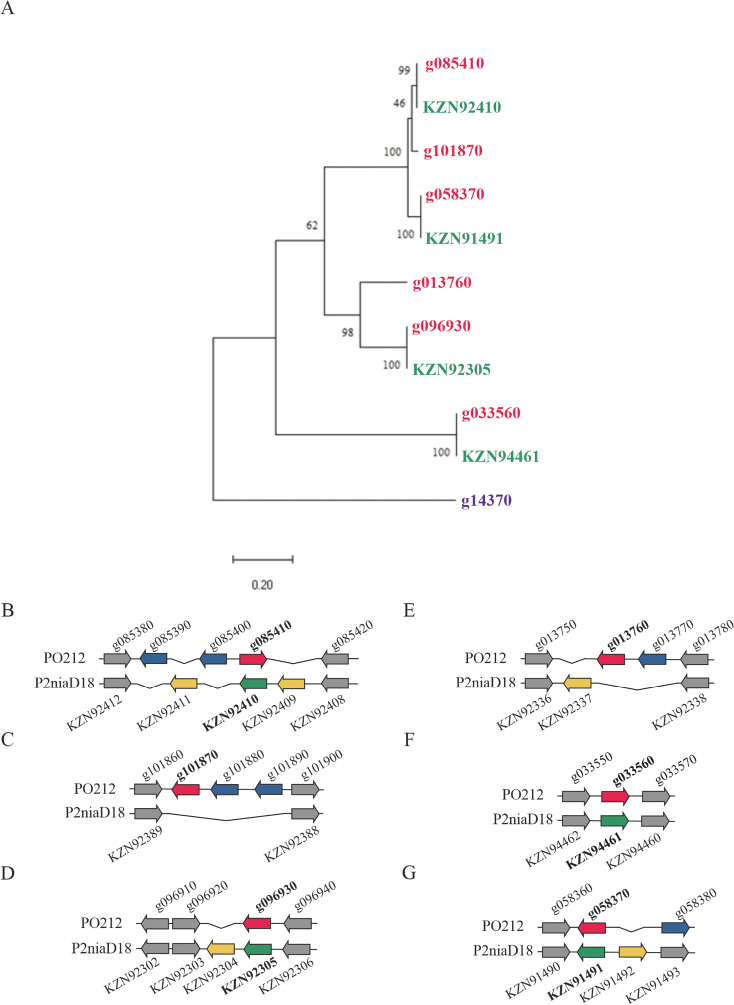
**A** Phylogenetic relationship of the genes encoding mutanases from PO212 and P2niaD18 using MEGA11. The phylogenetic tree was built using six mutanases encoding genes from PO212 (PO212g058370, PO212g085410, PO212g101870, PO212g096930, PO212g013760 and PO212g033560) (red) and the corresponding genes encoding mutanases from P2niaD18 (KZN91491, KZN92410, KZN92305 and KZN94461) (green). The sequence of a glycosyl transferase was utilised as a root (PO212g014370) (purple). Bootstrap values are indicated adjacent to the nodes. The tree was inferred by Maximum Likelihood. The tree has been drawn to scale, with branch lengths measured as the number of substitutions per site. The codon positions encompass in the analysis included first+second+third+non-coding positions. The final dataset comprised 4060 positions; **B** schematic representation of PO212g085410 mutanase and the homologue in P2niaD18 (KZN92410); **C** PO212g101870 mutanase and the homologue region in P2niaD18; **D** PO212g096930 mutanase and the homologue in P2niaD18 (KZN92305); **E** PO212g013760 mutanase and the homologue region in P2niaD18; **F** PO212g033560 and the homologue in P2niaD18 (KZN94461); **G** PO212g058370 and the homologue in P2niaD18 (KZN91491). The flanking and homologous genes between PO212 and P2niaD18 are coloured in grey. Red colour indicates PO212 mutanases. Blue colouration indicates the single genes for PO212. P2niaD18 mutanases are represented in green colour. The genes that are unique to P2niaD18 are shown in yellow. The prefix PO212 has been removed from the nomenclature of the genes in order to facilitate the legibility of the figure. The genes are to be initiated with the letter “g”.

Using the CAZymes of PO212 and P2niaD18 as a means to examine genetic variability, a comparison was made between these protein sets in the two strains (Suppl. material 5). This analysis revealed a greater number of differences when compared with the comparison between PO212 and S27. These differences were analysed in more detail (Suppl. material 7). The present study focuses on three of these differences. Firstly, the searches found 11 enzymes of the Auxiliary Activity (AAs) family in P2niaD18 without correspondence in PO212. Amongst these 11 enzymes, we identified two enzymes associated with conidial pigment biosynthesis, one laccase-1, one chitin-binding type-4 domain-containing protein, one putative FAD-linked oxidoreductase, one uncharacterised protein and five lytic polysaccharide mono-oxygenases. The second discrepancy pointed to the presence of six gene models in PO212 encoding alpha-(1, 3)-glucanases family members, also termed mutanases, while, in P2niaD18, only four of these enzymes were identified. The phylogenetic tree derived from the coding sequences of mutanases revealed different clades containing putative homologs between PO212 and P2niaD18 (PO212g085410-KZN92410, PO212g058370-KZN91491, PO212g096930-KZN92305 and PO212g033560-KZN4461) (Fig. 5A). The arrangement of the loci, incorporating the mutanase coding genes as well as flanking genes, is shown in Fig. 5B–G. Changes in the direction of the mutanase gene were observed between PO212 and P2niaD18 (Fig. 5B), as well as the presence of new predicted gene patterns for each of the strains (Fig. 5B–F). However, it was observed that PO212g101870 and PO212g013760 mutanases lacked a counterpart in P2niaD18 (Fig. 5C, E), despite their proximity to the clades formed by PO212g085410-KZN92410 and PO212g096930-KZN92305, respectively (Fig. 5A). As demonstrated in Fig. 5A, F, G, the PO212g058370 and PO212g033560 mutanases have their homologous genes in P2niaD18 and function as internal controls (Fig. 5A, F, G). Finally, the third discrepancy identified in the comparison of predicted CAZymes between PO212 and P2niaD18 revealed an early-truncated GT in the PO212 genome (PO212g072120). This gene is characterised by the absence of a T in its nucleotide sequence. The GeMoMa-based gene model of PO212g072120 suggested the presence of an intron thereby circumventing this early stop codon. However, amino acid sequence comparisons amongst putative homologues found in databases showed that the predictor generated an internally deleted sequence to prevent an early stop in this GT member. As was the case in previous comparative analysis, the same variation was found in S27 (S27g096810). This comparison reflects the existence of variations in other *P.rubens* genomes and demonstrates the close similarity of the PO212 and S27 strains, subsequent to the discarding of the predicted differences considered to be artefacts.

The incorporation of the CAZymes proteins of P2niaD18 in the analysis demonstrated the high conservation amongst the proteomes of the strains and further reinforced the strong resemblance between PO212 and S27 strains (Suppl. materials 5–7).

### ﻿Single nucleotide variants in coding regions of PO212 and S27

The search for differences between these two very similar strains, PO212 and S27, continues by the detection of single nucleotide variations (SNVs). For this, short (Illumina) and filtered reads (reads left after removing adapters, filtering by quality, removing duplicates and filtering by length) from S27 were mapped to the final version of the PO212 genome. We detected 161 variations between the two genomic sequences. Of these, 125 were localised in non-coding regions. Of the 36 variations in ORFs, only 25 would predictably cause an amino acid change. The frequency of nucleotide changes (the number of times a base is represented at a given position, with respect to the total number of reads that map to that position) in these 25 variations ranged from 65.31% to 100%. Of these, the frequency of the occurrence of the nucleotide alteration was found to be 100% in 10 of the variations and 98.95% in the remaining one.

The same results were obtained when the reverse analysis was performed, mapping the PO212 reads to the S27 reference genome. Confirmation was thus provided for the presence of six of the 11 variations already described in a previous study (Table 4). Sanger sequencing confirmed the five new variations found in PO212. Additionally, these five loci were also sequenced in alternative isolates of *P.rubens* with and without BA (Table 5). SNVs were identified in PO212g031060, which encodes an acyl-CoA desaturase; PO212g052230 that encodes a polarised growth protein; PO212g055060, which encodes an mRNA export factor; PO212g063880, which encodes a putative NADP-dependent oxidoreductase; and PO212g077520 that encodes a hypothetical protein (Table 5). It is noteworthy that three of these five genes (PO212g031060, PO212g055060 and PO212g063880) exhibited variations exclusively in PO212, while for the remaining two genes (PO212g052230 and PO212g077520) demonstrated variations in conjunction with CH6 and CH16. It was determined that these variations were not implicated in the biocontrol phenotype, as they were not observed in all those strains exhibiting BA (Table 5).

**Table 4.** Variations showed a 100% frequency of the single nucleotide variants (SNVs) between the PO212 and S27 genomes.

Code in PO212	Code in S27	Triplet PO212/S27 (5’- 3’)	Amino acid change
PO212g022060	S27g022120	**T**GC/**G**GC	Cys605Gly^†^
PO212g031060	S27g031140	**G**TC/**A**TC	Val354Ile
PO212g034660	S27g033250	**G**GT/**A**GT	Gly135Ser^†^
PO212g041380	S27g039990	**C**GA/**T**GA	Arg327^†, ‡^
PO212g046560	S27g045170	C**C**T/C**A**T	Pro219His^†^
PO212g052230	S27g050830	C**T**G/C**C**G	Leu283Pro
PO212g052600	S27g051200	G**A**T/G**G**T	Asp1447Gly^†^
PO212g055060	S27g053660	C**A**C/C**G**C	His949Arg
PO212g063880	S27g062120	**T**GC/**G**GC	Cys86Gly
PO212g077520	S27g081900	**A**CT/**G**CT	Thr19Ala
PO212g079090	S27g083470	**T**CC/**C**CC	Ser271Pro^†^

^†^Variations already shown as differences between PO212 and S27 genomes in Requena et al. 2023a. ^‡^Early stop in the coding sequence.

**Table 5.** Genotyping of five loci in the strains of the INIA-CSIC collection.

Strain	BA ^†^	g031060^§^	g052230^§^	g063880^§^	g055060^§^	g077520^§^
**PO212**	+	G	T	T	A	A
**S27**	-	A	C	G	G	G
**S17**	-	A	C	G	G	G
**S71**	-	A	C	G	G	G
**S73**	+	A	C	G	G	G
**CH2**	-^‡^	A	C	G	G	G
**CH5**	-^‡^	A	C	G	G	G
**CH6**	+^‡^	A	T	G	G	A
**CH8**	+^‡^	A	C	G	G	G
**CH16**	+^‡^	A	T	G	G	A

^†^BA: Biocontrol activity. ^‡^Unpublished results, general screenings of BA of strains INIA-CSIC. The symbol + indicates biocontrol activity. The symbol – indicates no biocontrol activity. ^§^ The prefix PO212 has been removed from the nomenclature of the genes in order to facilitate the legibility of the figure.

### ﻿Searching for mobile and repetitive elements

**Figure 6.**
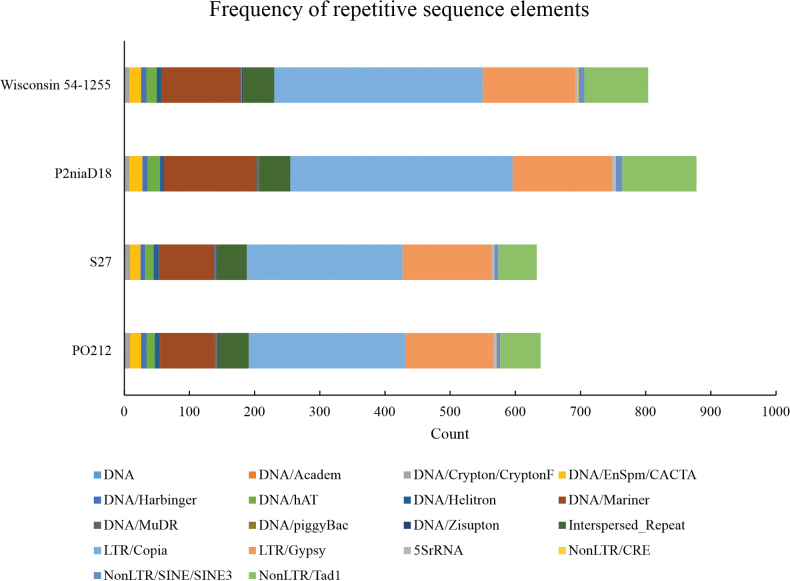
Frequency of repetitive sequence elements contained in the assemblies of Wisconsin 54-1255, P2niaD18, PO212 and S27.

Following a thorough analysis of the structural genome organisation, which revealed elevated levels of structural conservation and the absence of significant rearrangements, a repetitive sequence analysis was conducted on the PO212 and S27 assemblies. These results were then compared with a similar analysis of the P2niaD18 and Wisconsin 54-1255 assemblies (Fig. 6). The results demonstrate that the PO212 and S27 assemblies exhibit a comparable number of repetitive sequence elements (Fig. 6; Suppl. materials 8, 9) compared to the repetitive elements of P2niaD18 and Wisconsin 54-1255, which have a significant higher number of these sequences (Fig. 6; Suppl. materials 10, 11). The distribution of repetitive elements along the genomes in PO212, S27, P2niaD18 and Wisconsin 54-1255 is demonstrated in Fig. 2 and Fig. 3C, D, where it is observed that all scaffolds contain repetitive elements, with a higher number of elements in some specific regions.

### ﻿Presence of two Numts in the genomes of *P.rubens* strains

**Figure 7.**
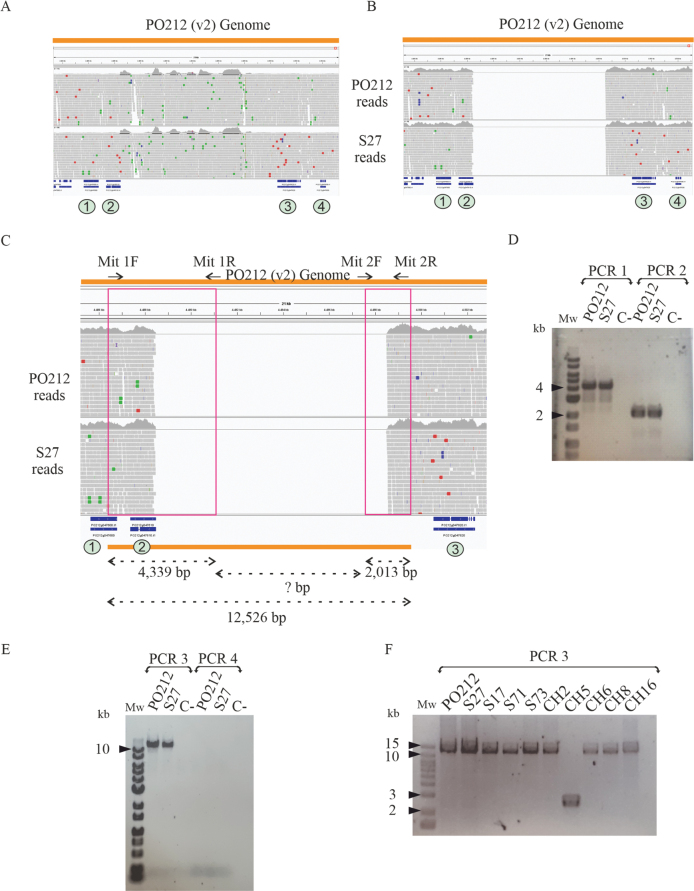
Identification of Numts in *Penicilliumrubens*: **A**PO212 and S27 raw reads were mapped to the PO212 genome in scaffold 2 between coordinates 4,488,480–4,498,480; **B** reads from PO212 and S27 sequencing that did not map to the mitochondrial genome of P2niaD18, were mapped to the PO212 genome in scaffold 2 between coordinates 4,488,480–4,498,480; **C** schematic representation illustrates the oligonucleotides mapping and predicted amplicon size. Green circles indicate the gene number: 1- PO212g047600, 2- PO212g047610, 3- PO212g047620, 4- PO212g047630. IGV images; **D** the PCR product of PCR 1 (Mit 1F and Mit 1R) and PCR 2 (Mit 2F and Mit 2R). The amplified fragment is 4.3 kb for PCR 1 and 2 kb for PCR 2, corresponding to the predicted size of the amplicon in both strains, PO212 and S27. C- Corresponds to the negative control of the PCR. Mw: Molecular weight marker; **E** PCR product of PCR 3 (Mit 1F and Mit 2R) and PCR 4 (same oligonucleotides as PCR 3). The amplified fragment for PCR 3 is greater than 10 kb for both strains, PO212 and S27. No PCR product was detected in PCR 4. C- Corresponds to the negative control of the PCR. Mw: Molecular weight marker; **F** PCR product of PCR 3 in other *P.rubens* DNA strains, where an amplicon greater than 10 kb can be observed for all strains except CH5 (2.5 kb).

Two regions with a very high number of reads were found when mapping the PO212 and S27 reads to their own assemblies (Fig. 7A and Suppl. material 1: fig. S1A). Thus, while the coverage in random regions of the genome is 60x depth, in the aforementioned regions where reads accumulate, the coverage reaches an average of 750x. The initial hypothesis concerning the nature of these read accumulations was that they belonged to repetitive regions. However, subsequent meticulous scrutiny of the sequences revealed the presence of mitochondrial DNA segments within genomic regions. These regions were previously described as Numts (nuclear mitochondrial DNA segment). In this study, two such Numts were identified in several *P.rubens* isolates, including PO212 and S27.

In order to verify the mitochondrial nature of these two regions, the sequencing reads from both, the PO212 and S27 strains, were mapped to the P2niaD18 strain mitochondrial genome (GCA_000710275.1) as the best-assembled genome of the *P.rubens* clade. Reads that failed to map to the P2niaD18 mitochondrial genome were subsequently mapped to the PO212 genome. Following the elimination of mitochondrial reads, two gaps, one measuring 10 kb and the other 500 bp, were identified in the alignment of the reads to the PO212 assembly (Fig. 7B and Suppl. material 1: fig. S1B). A similar analysis performed with the S27 assembly revealed similar gaps when mapping non-mitochondrial reads. The presence of such mitochondrial sequences in the nuclear genome was verified by means of PCR analysis (Fig. 7C). The same strategy was employed for both gaps. For the largest gap (10 kb), an initial investigation was conducted to ascertain the presence of amplification using Mit 1F and Mit 1R oligonucleotides (PCR 1) and Mit 2F and Mit 2R (PCR 2). The results of these PCRs yielded an amplicon of 4.3 kb and 2 kb, respectively (Fig. 7D). Two further PCRs (PCR 3 and PCR 4) were designed to amplify the internal fragment between the Mit 1F and Mit 2R oligonucleotides, with a length of 12.5 kb over the genome sequence. The third PCR programme (PCR 3) was characterised by an amplification time sufficient to amplify a fragment greater than 12.5 kb, in contrast to the shorter amplification time employed in the fourth PCR programme (PCR 4). The results demonstrated an amplicon greater than 10 kb in PCR 3 (longest amplification), but no PCR product was detected in PCR 4 (Fig. 7E). Subsequent to this, the 10 kb fragment was subjected to sequencing (PCR 3) using Mit 1R and Mit 2R oligonucleotides. This confirmed its alignment with the mitochondrial sequence, thereby validating the insertion of a 10 kb mitochondrial region into nuclear DNA. Suppl. material 2 illustrates the correspondence between the mitochondrial sequence that was inserted into the PO212 and S27 genomes and the Wisconsin 54-1255 mitochondrial genome.

This largest Numt (10 kb) is found in PO212 and S27. In PO212, it is located in scaffold 2 between coordinates 4,488,480–4,498,480 and, in the S27 assembly, between coordinates 4,048,300–4,058,300 of scaffold 2. The flanking genes of this region are the PO212g047600 (1) and PO212g047610 (2) genes on one side and the genes PO212g047620 (3) and PO212g047630 (4) on the other side. The equivalent flanking genes in S27 are S27g046190 and S27g046200 on one side, S27g046210 and S27g046220 on the other side. Due to the proximity of the flanking gene PO212g047610 and its homologue in S27 (S27g046200) to the inserted mitochondrial region, putative homologues were identified in P2niaD18 (KZN83398) and Wisconsin 54-1255 (Pc16g00290). The PO212g047610, S27g046200 and Pc16g00290 gene models, predicted a different translation start site than KZN83398. In the latter, the ATG is predicted to occur at an upstream position, resulting in the addition of 103 amino acids to the expected protein sequence encoded by the remaining three loci. Analysis of putative homologues in other *Penicilli* gives support to the hypothesis that this second ATG could also function as a translation initiation site for these gene models.

The second Numt of 500 bp detected is located on scaffold 5, between coordinates 3,232,580–3,233,080 in the PO212 assembly. Genes PO212g085220 (5) and PO212g085230 (6) are located on one side of the Numt, whilst PO212g085240 (7) and PO212g085250 (8) are located on the other side (Suppl. material 1: fig. S1C). In the case of S27, this Numt is located on scaffold 6 between coordinates 5,618,555–5,619,055 with the flanking genes being S27g089590 and S27g089600 on the left side and S27g089610 and S27g089620 on the right side. The utilisation of a PCR and the subsequent sequencing of this region confirmed the presence of this mitochondrial region inserted into nuclear DNA (Suppl. material 1: fig. S1D).

The presence of these two Numts was verified by PCR in other *P.rubens* strains used in this study (Table 1). With the exception of CH5, all strains exhibited an amplicon larger than 10 kb when using Mit 1F and Mit 2R oligonucleotides (Fig. 7F). The PCR 3 fragment using genomic DNA from the CH5 strain matched the predicted 2.5 kb genomic region lacking the mitochondrial insert. PCR 5 (Mit 3F and Mit 3R oligonucleotides) was performed to amplify the smallest Numt. The amplicon size for all strains corresponded to the expected size for this region (2.5 kb), with the exception of the CH2 strain, which did not yield any PCR product (Suppl. material 1: fig. S1E).

**Figure 8.**
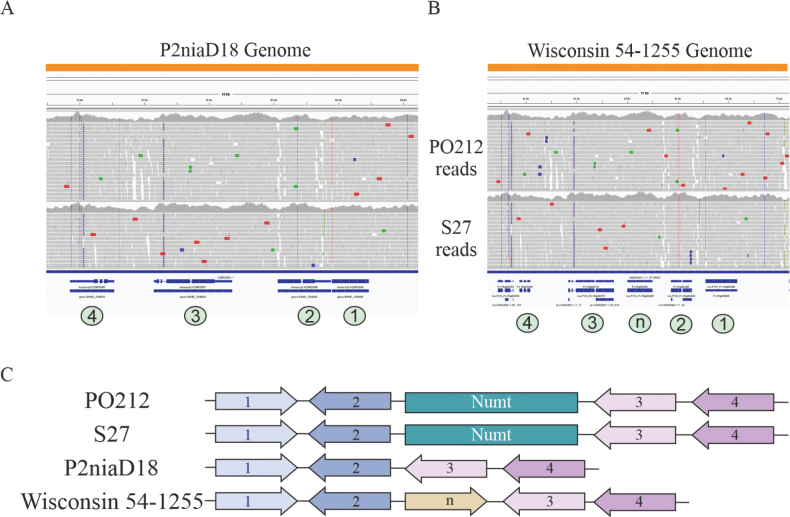
Coverage of PO212 and S27 reads on the P2niaD18 and Wisconsin 54-1255 assemblies: **A** alignment of PO212 and S27 reads to the P2niaD18 genome, on chromosome IV spanning coordinates 71,228–85,548. The gene number is indicated by the colour green (from left to right): 4- KZN83396, 3- KZN83397, 2- KZN83398, 1- KZN83399; **B**PO212 and S27 reads mapped to the Wisconsin 54-1255 genome in AM920431.1:55,537–73,456. Green circles are used to indicate the gene number (from left to right): 4- Pc16g00260, 3- Pc16g00270, n- Pc16g00280, 2- Pc16g00290 and 1- Pc16g00300. IGV images; **C** schematic representation of the organisation of this region in the PO212, S27, P2niaD18 and Wisconsin 54-1255 genomes.

Following the confirmation of the presence of Numts in the PO212 and S27 genomes, the P2niaD18 and Wisconsin 54-1255 genomes were subjected to further analysis. However, due to the unavailability of these strains in the laboratory, it was not possible to carry out PCR analysis. Consequently, a genome check was performed as an alternative. For this approach, unmapped PO212 and S27 reads with the P2niaD18 mitochondrial genome, were mapped to the P2niaD18 and Wisconsin 54-1255 genomes. As demonstrated in Fig. 8A, B, the PO212 and S27 reads exhibit uniform coverage of this region. This finding suggests the absence of a Numt in this region in the P2niaD18 and Wisconsin 54-1255 assemblies. It is noteworthy that the genome of Wisconsin 54-1255 contains an additional gene, designated Pc16g00280 and labelled “n”, which is positioned between the genes labelled as 2 and 3 (Fig. 5B, C). No evidence for this pseudogene (van den Berg et al. 2008) was found in either the PO212 or S27 assemblies.

## ﻿Discussion

The advent of sequencing technologies (Zimin et al. 2017), such as PacBio and Illumina, has enabled the generation of hybrid assemblies that exhibit a degree of quality sufficient to facilitate more comprehensive subsequent predictions. The aim of this study was to analyse the genome organisation and the presence of repetitive sequences as transposable elements (TEs), as well as to study genetic variability between strains PO212 and S27 by predicting a large protein family as the CAZymes. The employment of long read sequencing and assembly procedures (automatic and manual) have culminated in the generation of two genomes, each comprising 13 scaffolds. Currently, 102 genomes are available in the NCBI databases (https://www.ncbi.nlm.nih.gov/datasets/genome/?taxon=1108849,5076,3062267) between the *P.rubens* and *P.chrysogenum* clades. The preponderance of these genomes is characterised by their organisation into a minimum of more than 100 scaffolds. However, it is notable that 11 genomes exhibit genome organisation into 15 or fewer scaffolds (Specht et al. 2014; Petersen et al. 2023).

In a previous study (Requena et al. 2023a), draft versions of the genomes of strains PO212 and S27 using Illumina sequencing were presented and compared. PO212 is a biocontrol agent that reduces vascular wilt of tomato caused by Fusariumoxysporumf.sp.lycopersici (De Cal et al. 1995). In contrast, S27 lacks such BA (Requena et al. 2023a). In that study, the PO212 genome was organised into 65 scaffolds while the S27 genome was organised into 414 scaffolds (Requena et al. 2023a). The study demonstrated the strong sequence conservation between these two draft genomes, although the excessive number of scaffolds, into which the assemblies were arranged, prevented a higher degree of assembly and required an additional finishing step. Current hybrid assemblies are organised into a reduced number of scaffolds, enabling studies of genomic organisation that are more reliable, as well as changes that may affect biological processes of interest, caused by the integration of endogenous or exogenous DNA.

The genome organisation presented in the synteny diagram between PO212 and S27 assemblies does not show any reorganisations between the genomes, such as translocations or inversions within scaffolds. The breakpoints observed between the two genomes could not be joined due to the parameters imposed during the assembly procedures. Regions exhibiting low read coverage were found to be inadequate in providing sufficient evidence for the closure of gaps between scaffolds. However, further analysis in these regions confirmed that there was no loss of information between the two assemblies. By contrast, a high degree of genome rearrangement is observed when the PO212 assembly is compared with those of P2niaD18 and Wisconsin 54-1255. This high degree of rearrangement is likely linked to the mutagenesis process used to enhance penicillin production (Specht et al. 2014). P2niaD18 and Wisconsin 54-1255 have already been described as having chromosome rearrangements affecting chromosomes II and III and chromosomes III and IV, while both are descendants of the wild-type strain *P.chrysogenum* NRRL 1951 (Specht et al. 2014). The high quality of the assembly in P2niaD18 and the extensive use of the Wisconsin 54-1255 assembly as a reference genome amongst *Penicillium* by both the present authors and their colleagues have guided the selection of these genomic assemblies.

With regard to the presence of repetitive sequences and TEs, the comparison between PO212 and S27 analysis demonstrated minimal disparities, suggesting a high degree of similarity in the quantity of TEs between the two strains under study. However, a greater discrepancy was observed in the predictions of P2niaD18 and Wisconsin 54-1255. This discrepancy can be attributed, at least in part, to the geographical origins of the industrial strains. The distribution of TEs seems to be homogeneously dispersed along the scaffolds, despite the existence of varying levels of preference for their insertion within the genome, as highlighted by Bourque et al. (2018). The distribution of TEs throughout the genome may result in different levels of gene expression. It is increasingly evident that TEs can alter the gene expression of nearby genes, as postulated by McClintock (1956) and as supported by numerous studies referenced in the review by Bourque et al. (2018).

In the context of ongoing research investigating the potential for endogenous DNA to undergo modification, the Numts were identified. Sequencing from long reads, as enabled by the PacBio’s technology and not removing the mitochondrial reads before assembling the genomes, enable the detection of Numts, defined as segments of mitochondrial DNA that have been inserted into the nuclear genome (López et al. 1994). These Numts were previously undescribed in the genus *Penicillium*. It is important to note that the order in which the nuclear and mitochondrial genomes are assembled affects the identification of Numts in genomes. As demonstrated in Fig. 7B, the removal of mitochondrial reads prior to the assembly of the nuclear genome has been shown to prevent the assembly of genomic regions containing Numts. Indeed, these Numts were detected when different alignments of raw sequences were performed. The elevated number of reads encompassing the regions harbouring the Numts has instigated the hypothesis of potential gene duplications or elevated copy numbers. Direct sequencing of flanking regions of these Numts confirmed their presence in either the PO212 or S27 assemblies, as well as in other strains from the laboratory collection. As previously highlighted by other authors (Li et al. 2021), the observation of Numts in the same localisation in PO212, S27 and other strains used in this study, provides substantial evidence for the integration of these Numts. The presence of Numts has previously been observed in species of other kingdoms (Lopez et al. 1994; Noutsos et al. 2005; Pamilo et al. 2007; Sacerdot et al. 2008; Trian and DeWoody 2008; Hazkani-Covo et al. 2010; Li et al. 2021). Two biocontrol strains of *Trichoderma* have been found to harbour three Numts (Li et al. 2021). To the best of our knowledge, however, no Numts have been identified in the genus *Penicillium*, to date. All strains of *P.rubens* from the collection utilised in this study (Villarino et al. 2016; Espeso et al. 2019; Requena et al. 2023a) possessed both Numts with the exception of two strains, CH2 and CH5, which contained one Numt insertion each. The origin of the strains does not appear to influence the presence of the Numts, as all strains are from different sites, including soil samples, pruning shoots, shoots and leaves of perennial plants. Extending this analysis to encompass a greater number of strains could yield additional insights into the prevalence of these insertions in *Penicillium* genomes and the integration of mitochondrial sequences into the nuclear genome. In a manner analogous to the mobility of TEs, the integration of Numts can also be subject to alteration of coding genes. In this respect, comparative analysis with sequences from other strains belonging to the same clade did not demonstrate that the Numts insertion disrupted any coding regions. An important feature of the PO212 strain is that it exhibits BA. Nevertheless, the presence of these insertions does not appear to be a determining factor in this particular phenotype, as these insertions have also been observed in other strains that do not exhibit this activity.

A prediction of the CAZymes was made from the current assemblies. This group is quite large and can provide insight not only into the annotation of carbohydrate-active enzymes, but also into the strong resemblance between the two strains studied. The search for discrepancies between the predictions of the two assemblies returned no results after the verification of the artefacts by the predictors. In this study, we analysed some differences that we have identified as artefacts between PO212 and S27. These predictions are essential for analysing an organism’s capacity for encoding different proteins. Nevertheless, it is imperative to verify the automated predictions. Failure to review such predictions can lead to the accumulation of errors in subsequent processes (Promponas et al. 2015). In the present case, the absence of significant disparities in CAZymes prediction is congruent with the genomic similarity observed between these strains. Several artefacts were found at the genomic level. These were discarded. The most prevalent artefacts identified in the comparison between PO212 and S27 included the absence of gene models due to failure to assemble small genomic regions, where further analysis using raw sequences verified their presence (Suppl. material 3). Secondly, the generation of gene models harbouring artefactual introns was undertaken to prevent the presence of stop codons in the predicted ORF (PO212g072120 and S27g096810). Thirdly, a failure to assign the most likely start codon in signal peptide predictions. The identification of discrepancies categorised as artefacts underscores the extensive and meticulous manual curation necessary to circumvent potential errors that could accumulate and affect other levels of prediction. A greater number of discrepancies in CAZymes were identified during the comparison of PO212 with P2niaD18. The differences found in this comparison further underscore the pronounced similarity between PO212 and S27 strains, given that these differences are also exhibited by S27. However, as previously mentioned, caution must be exercised when interpreting these differences, as they may be artefacts.

A slight increase in the number of predicted genes was observed in comparison with previous assemblies, which may be attributable to a reduced fragmentation of the genomes by long read sequencing (Requena et al. 2023a). This increase in new sequencing reads has facilitated the detection of five single nucleotides variants (SNVs). These variations can be considered in the future as possible targets for the design of oligonucleotides that would allow this strain to be differentiated. However, it is crucial to emphasise that this assertion requires further validation thorough the accumulation of sequencing data. The analysis of these variations, in conjunction with the previously identified ones, was discarded to be responsible for the BA of PO212. However, the remaining variations, located in intergenic regions, could play a fundamental role in the expression of genes that could be involved in the biocontrol phenotype of PO212. Differences at the transcriptional level between PO212 and S27 have already been observed for the transcription factor XlnR (Requena et al. 2023b). These findings suggest the possibility of divergence responses to specific processes between strains. Consequently, subsequent studies will concentrate on investigating the potential consequences of intergenic variations.

## ﻿Conclusions

The employment of long sequence technologies and assembly work have facilitated the acquisition of two assembled genomes of *P.rubens* strains, thereby augmenting the compendium of genomes stored in the databases. The PO212 and S27 assemblies are distinguished by their remarkable genomic conservation, exhibiting no discernible structural rearrangements, despite the spatial and temporal variations in the strains obtained from soil samples. The analysis of these assemblies revealed the presence of transposable elements dispersed throughout the genome, thereby underscoring the genetic similarity between the two study strains. In addition, the presence of mitochondrial sequences inserted into the nuclear genome of both strains was observed. The integration of these sequences appears to be a unique event, as these and other strains analysed show integration in the same region of the genome, being the first time this has been observed in the genus *Penicillium*. Extending this analysis to other strains could provide further insights into the insertion process of these mitochondrial sequences into the nuclear genome. On the other hand, the CAZymes prediction highlights the remarkable similarity of these two strains with different activity in the biological control of plant diseases and underlines the need to verify the differences. Further analysis including transcriptomic studies and intergenic variations, would enhance our understanding of the BA of PO212.

## ﻿Additional information

### Adherence to national and international regulations

All the fungal strains used in this study have been legally obtained, respecting the Convention on Biological Diversity (Rio Convention).

### Author ORCIDs

Elena Requena https://orcid.org/0000-0001-5028-8880

Javier Veloso https://orcid.org/0000-0002-7283-769X

Eduardo A. Espeso https://orcid.org/0000-0002-5873-6059

Inmaculada Larena https://orcid.org/0000-0001-8424-8916

### Data availability

The datasets generated for this study can be found in online repositories (https://www.ncbi.nlm.nih.gov). Nucleotide sequence data reported are available in the NCBI databases under submission numbers: JAPDLE000000000.2, JAPDLD000000000.2.

## Supplementary materials

Supplementary material 1 Identification of Numts in *P.rubens* Data type png https://binary.pensoft.net/file/1336991This dataset is made available under the Open Database License (http://opendatacommons.org/licenses/odbl/1.0/). The Open Database License (ODbL) is a license agreement intended to allow users to freely share, modify, and use this Dataset while maintaining this same freedom for others, provided that the original source and author(s) are credited.Elena Requena, Javier Veloso, Eduardo A. Espeso, Inmaculada Larena

Supplementary material 2 Identification of inserted mitochondrial regions into genomic DNA Data type png https://binary.pensoft.net/file/1336992This dataset is made available under the Open Database License (http://opendatacommons.org/licenses/odbl/1.0/). The Open Database License (ODbL) is a license agreement intended to allow users to freely share, modify, and use this Dataset while maintaining this same freedom for others, provided that the original source and author(s) are credited.Elena Requena, Javier Veloso, Eduardo A. Espeso, Inmaculada Larena

Supplementary material 3 Mapped reads of different parts of the PO212 and S27 genomes Data type png https://binary.pensoft.net/file/1336993This dataset is made available under the Open Database License (http://opendatacommons.org/licenses/odbl/1.0/). The Open Database License (ODbL) is a license agreement intended to allow users to freely share, modify, and use this Dataset while maintaining this same freedom for others, provided that the original source and author(s) are credited.Elena Requena, Javier Veloso, Eduardo A. Espeso, Inmaculada Larena

Supplementary material 4 Oligonucleotides designed in this work Data type xlsx https://binary.pensoft.net/file/1336994This dataset is made available under the Open Database License (http://opendatacommons.org/licenses/odbl/1.0/). The Open Database License (ODbL) is a license agreement intended to allow users to freely share, modify, and use this Dataset while maintaining this same freedom for others, provided that the original source and author(s) are credited.Elena Requena, Javier Veloso, Eduardo A. Espeso, Inmaculada Larena

Supplementary material 5 Prediction of PO212, S27 and P2niaD18 CAZymes using DIAMOND and HMMER databases Data type xlsx https://binary.pensoft.net/file/1336995This dataset is made available under the Open Database License (http://opendatacommons.org/licenses/odbl/1.0/). The Open Database License (ODbL) is a license agreement intended to allow users to freely share, modify, and use this Dataset while maintaining this same freedom for others, provided that the original source and author(s) are credited.Elena Requena, Javier Veloso, Eduardo A. Espeso, Inmaculada Larena

Supplementary material 6 Comparison of CAZymes between PO212 and S27 genomes Data type xlsx https://binary.pensoft.net/file/1336996This dataset is made available under the Open Database License (http://opendatacommons.org/licenses/odbl/1.0/). The Open Database License (ODbL) is a license agreement intended to allow users to freely share, modify, and use this Dataset while maintaining this same freedom for others, provided that the original source and author(s) are credited.Elena Requena, Javier Veloso, Eduardo A. Espeso, Inmaculada Larena

Supplementary material 7 Comparison of CAZymes between PO212 and P2niaD18 genomes Data type xlsx https://binary.pensoft.net/file/1336997This dataset is made available under the Open Database License (http://opendatacommons.org/licenses/odbl/1.0/). The Open Database License (ODbL) is a license agreement intended to allow users to freely share, modify, and use this Dataset while maintaining this same freedom for others, provided that the original source and author(s) are credited.Elena Requena, Javier Veloso, Eduardo A. Espeso, Inmaculada Larena

Supplementary material 8 Repetitive elements for PO212 strain Data type xlsx https://binary.pensoft.net/file/1336998This dataset is made available under the Open Database License (http://opendatacommons.org/licenses/odbl/1.0/). The Open Database License (ODbL) is a license agreement intended to allow users to freely share, modify, and use this Dataset while maintaining this same freedom for others, provided that the original source and author(s) are credited.Elena Requena, Javier Veloso, Eduardo A. Espeso, Inmaculada Larena

Supplementary material 9 Repetitive elements for S27 strain Data type xlsx https://binary.pensoft.net/file/1336999This dataset is made available under the Open Database License (http://opendatacommons.org/licenses/odbl/1.0/). The Open Database License (ODbL) is a license agreement intended to allow users to freely share, modify, and use this Dataset while maintaining this same freedom for others, provided that the original source and author(s) are credited.Elena Requena, Javier Veloso, Eduardo A. Espeso, Inmaculada Larena

Supplementary material 10 Repetitive elements for P2niaD18 strain Data type xlsx https://binary.pensoft.net/file/1337000This dataset is made available under the Open Database License (http://opendatacommons.org/licenses/odbl/1.0/). The Open Database License (ODbL) is a license agreement intended to allow users to freely share, modify, and use this Dataset while maintaining this same freedom for others, provided that the original source and author(s) are credited.Elena Requena, Javier Veloso, Eduardo A. Espeso, Inmaculada Larena

Supplementary material 11 Repetitive elements for Wisonsin 54-1255 strain Data type xlsx https://binary.pensoft.net/file/1337001This dataset is made available under the Open Database License (http://opendatacommons.org/licenses/odbl/1.0/). The Open Database License (ODbL) is a license agreement intended to allow users to freely share, modify, and use this Dataset while maintaining this same freedom for others, provided that the original source and author(s) are credited.Elena Requena, Javier Veloso, Eduardo A. Espeso, Inmaculada Larena
