# Supplementary figures and images for: ﻿Hybrid assembly of Penicilliumrubens genomes unveils high conservation of genome structural organisation and the presence of Numts in nuclear DNA
Source: IMA Fungus. 2025 May 23;16:e145175. doi: 10.3897/imafungus.16.145175 (PMC12125605; doi:10.3897/imafungus.16.145175)

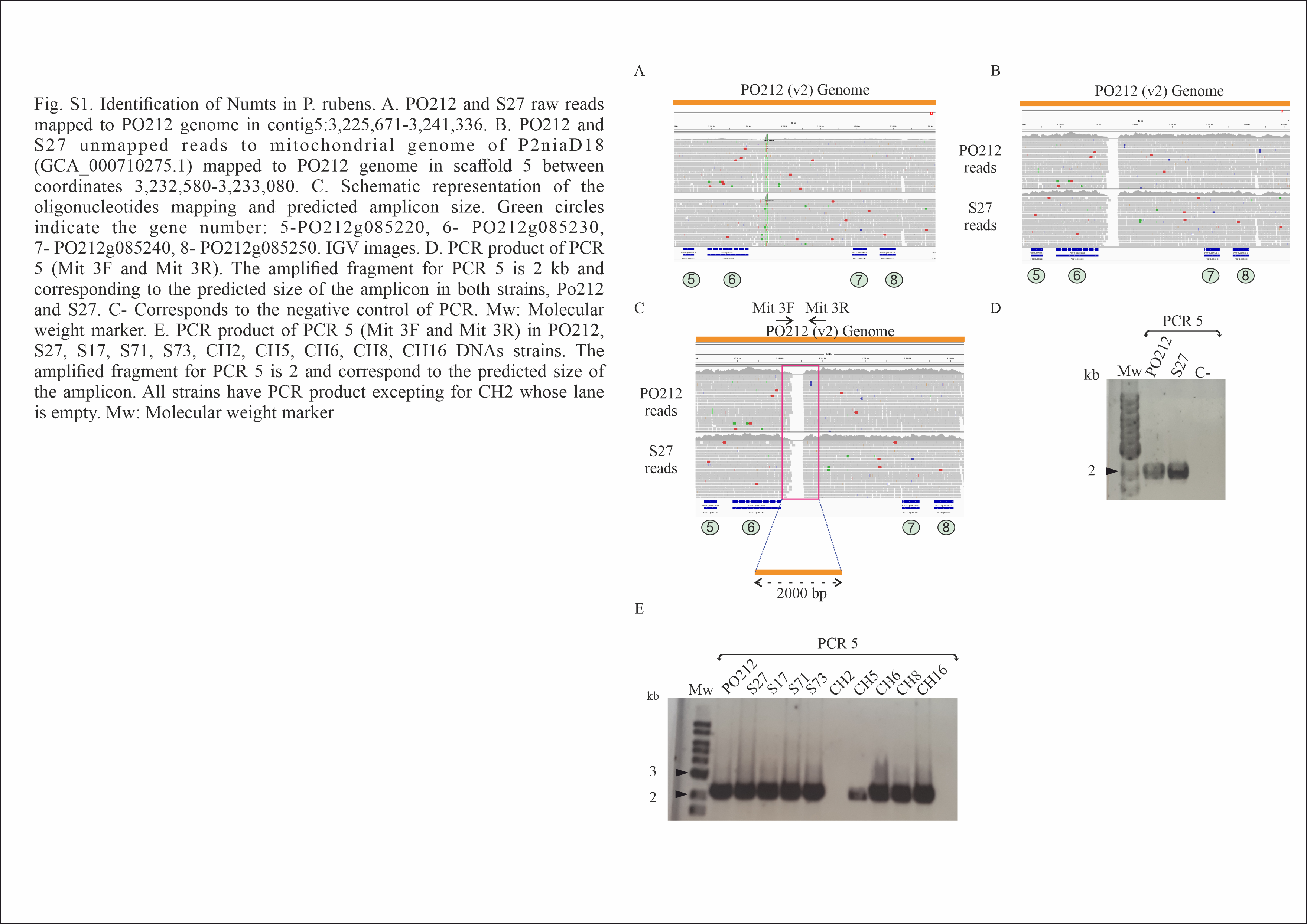

Supplement: Supplementary material 1 — Identification of Numts in P.rubens [file imafungus-16-e145175-s001.png]

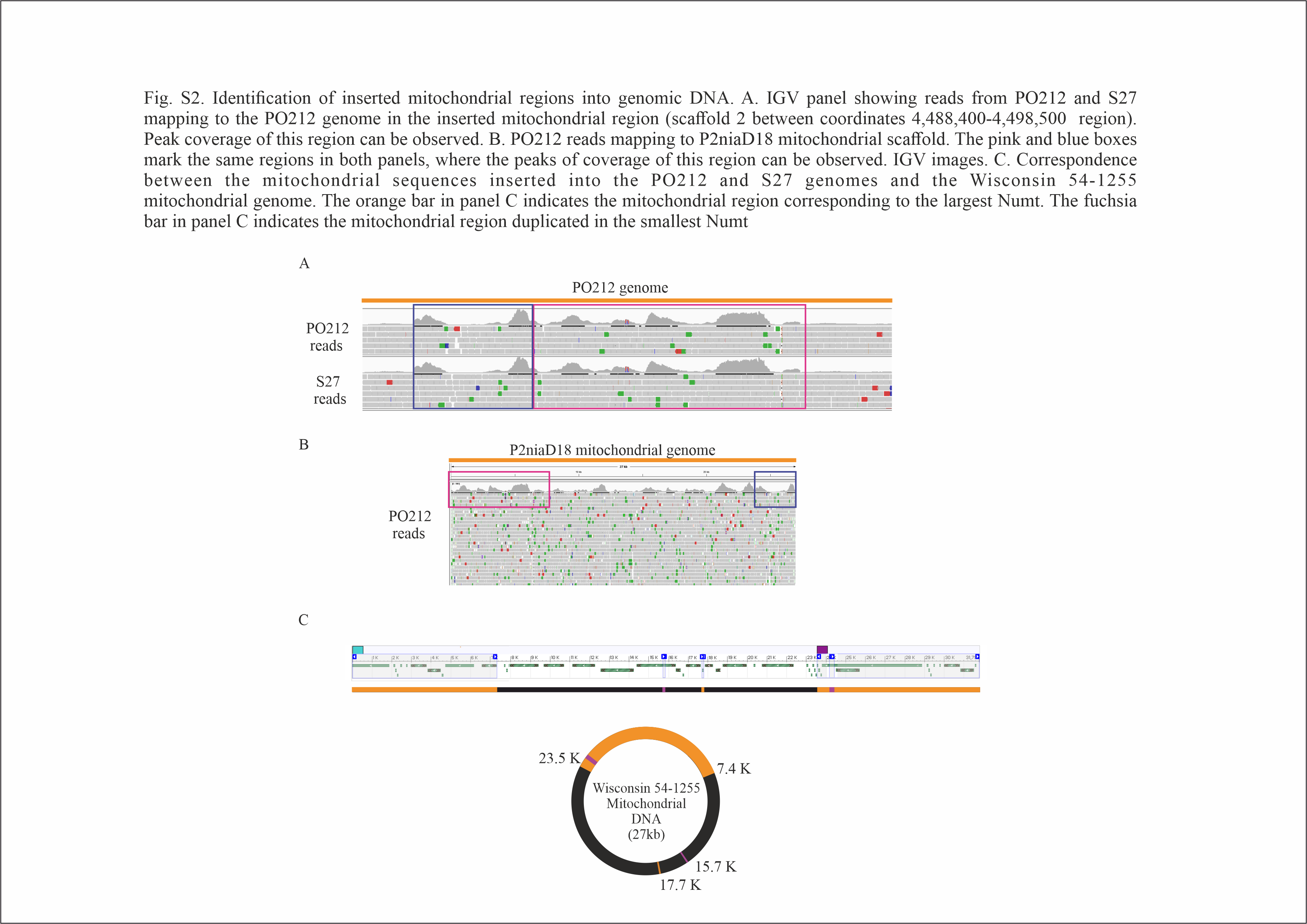

Supplement: Supplementary material 2 — Identification of inserted mitochondrial regions into genomic DNA [file imafungus-16-e145175-s002.png]

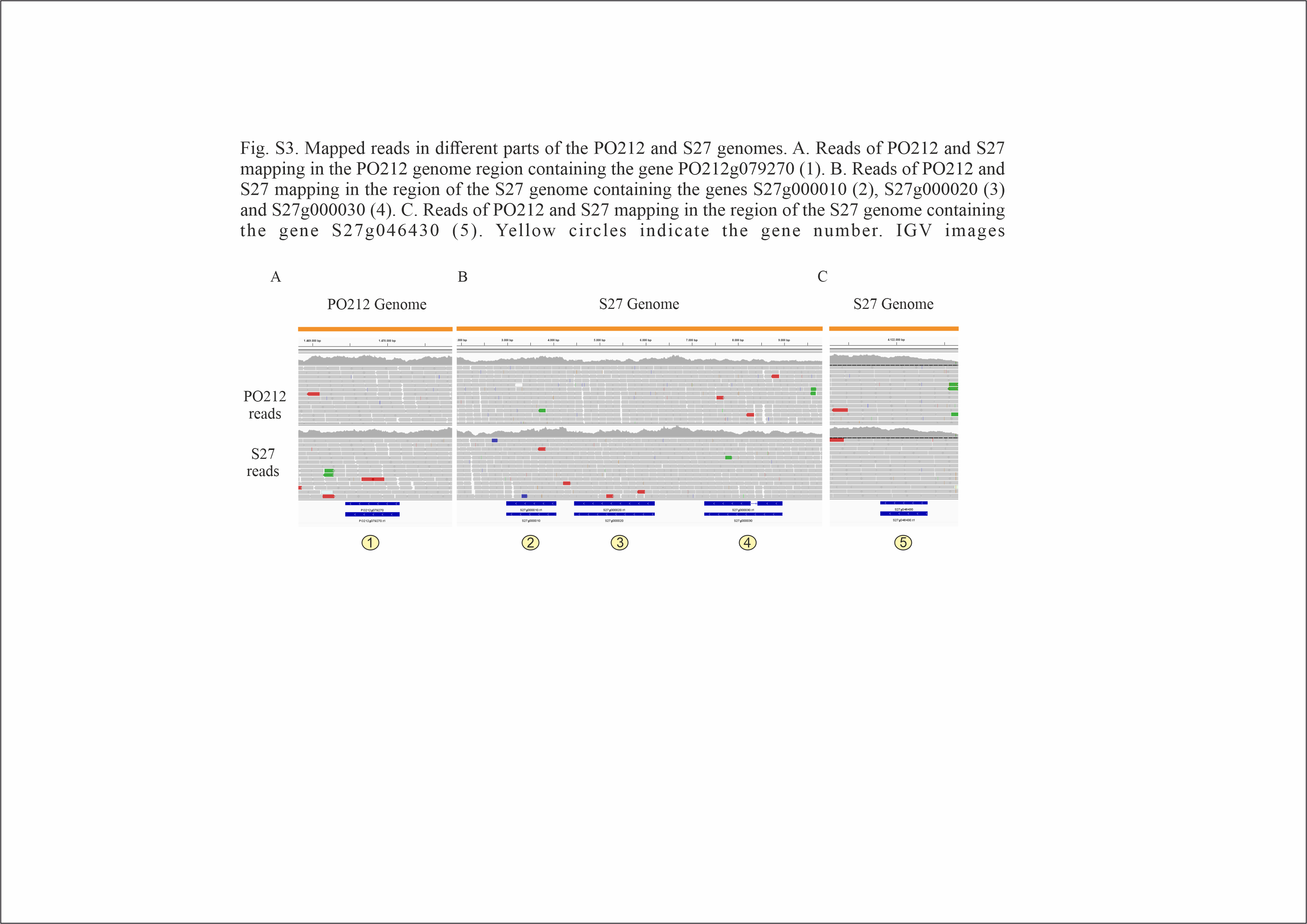

Supplement: Supplementary material 3 — Mapped reads of different parts of the PO212 and S27 genomes [file imafungus-16-e145175-s003.png]
